# The Editor's Letter-Box

**Published:** 1920-04-03

**Authors:** P. W. Kessler

**Affiliations:** Chairman of the Board of Management. Manchester Royal Eye Hospital, Oxford Road, Manchester


Sir,—While not approving the suggestion of a compulsory flat rate to be contributed in all cases, my Board is entirely in accord with the principle that hospital patients should contribute according to their means to the upkeep of the institution. As a matter of fact, this principle has been carried into practice for a number of years in this hospital as follows: Out-patients are invited to contribute to the "boxes" on the occasion of each visit. The amount thus collected from 38,000 patients in the year 1919 was £1,180. In-patients are asked to contribute towards the cost of their *maintenance* in the hospital; hitherto the amount asked for has been up to 10s. per week, but many have been unable to pay anything. For the year 1919, 1,662 in-patients contributed £320.

Bearing in mind the increase in the incomes of our patients, we are not satisfied that we are receiving from them the amounts that they should contribute; and we hope to improve upon the past. Under existing economic conditions it is important that all legitimate sources of income should be utilised.—Yours faithfully,

P. W. Kessler.

Chairman of the Board of Management.

Manchester Royal Eye Hospital, Oxford Road,
Manchester, March 25, 1920.

(*Continued on next page.*)

